# Conservative Management for Lingual Thyroid Ectopic

**DOI:** 10.1155/2015/265207

**Published:** 2015-02-15

**Authors:** Eder Alberto Sigua-Rodriguez, Douglas Rangel Goulart, Luciana Asprino, Afonso Celso de Moraes Manzano

**Affiliations:** ^1^Oral and Maxillofacial Surgery Division, Department of Oral Diagnosis, Piracicaba Dental School, State University of Campinas (UNICAMP), Campinas, SP, Brazil; ^2^Department of Head and Neck Surgery, Hospital Santa Casa de Limeira, Limeira, SP, Brazil

## Abstract

Lingual thyroid gland is a rare clinical entity. The presence of an ectopic thyroid gland located at the base of the tongue may be presented with symptoms like dysphagia, dysphonia, and upper airway obstruction. We are introducing a case of an 8-year-old girl who had lingual thyroid that presented dysphagia and foreign body sensation in the throat. The diagnostic was reached with clinical examination, thyroid scintigraphy with Tc^99m^ and ultrasound. A laryngoscopy was performed which confirmed a spherical mass at base of tongue. Investigation should include thyroid function tests. In this case we observed subclinical hypothyroidism. There are different types of surgical approaches for the treatment of this condition; however, the treatment with Levothyroxine Sodium allowed the stabilization of TSH levels and clinical improvement of symptoms in a follow-up of 2 years.

## 1. Introduction

Ectopic thyroid is an uncommon embryological abnormality characterized by the presence of thyroid tissue in a site other than its usual pretracheal location [1, 2]. Of all ectopic thyroids 90% are found to be lingual [3] It is a rare congenital anomaly appearing with prevalence of 1 : 100.000 [3].

Patients with lingual thyroid tissue usually present with symptoms such as dysphagia, choking, hemorrhage, and dyspnea and occasionally life-threatening airway obstruction. In addition, malignant transformation of the lingual thyroid has been reported, albeit rarely [4]. For patients with obstructive symptoms, thyroxine replacement should be introduced as initial therapy, to induce glandular shrinkage [5]. If conservative treatment fails, surgical removal is necessary [4].

Lingual thyroid is estimated to occur in 0.2 per cent of normal children, being more common in females and on the left side of the thyroid gland [1]. In this case report, the diagnosis and conservative treatment of this condition are highlighted.

## 2. Case Report

A child of eight years old came to our department with dysphagia and foreign body sensation in the throat. These symptoms were present to three months and showing greater intensity every day. She had no history of either past or present thyroid disease. After examination, the patient presented a solid, spherical mass with 2 cm of diameter, covered with intact mucosa, located at the base of the tongue. Examination of neck did not reveal cervical lymphadenopathy, the past medical history of the patient was insignificant, and she was not currently taking any medication.

**Figure 1:**
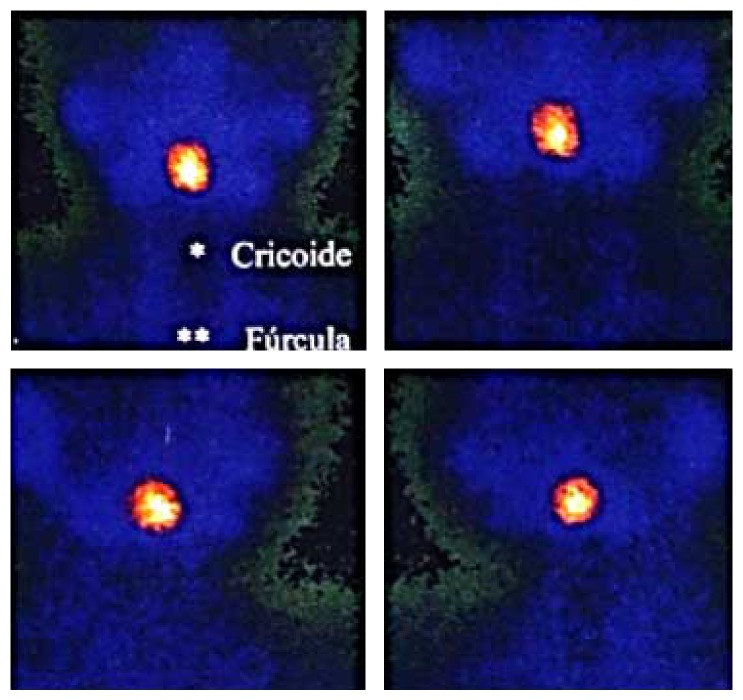
Scintigraphy—left side of neck. The quantification of radiopharmaceutical uptake by this change lies within the normal range of thyroid uptake.

**Figure 2:**
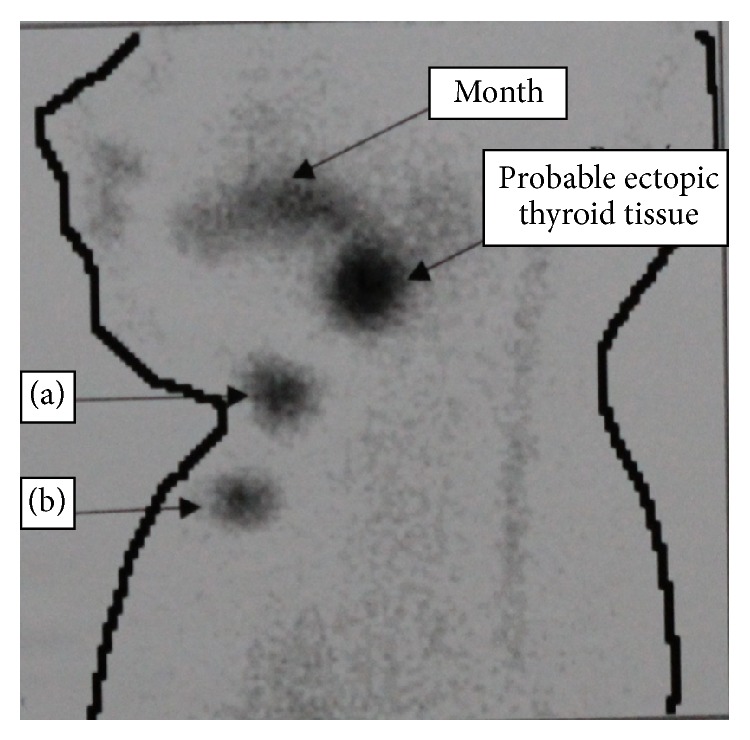
Scintigraphy interpretation. (a) Cricoid cartilage, (b) sternal furculum. Absence of uptake in the thyroid bed (anterior neck) with probable location at the base of the tongue, suggestive of ectopic thyroid.

Thyroid scintigraphy with Tc^99m^ and ultrasound were performed, which confirmed oval mass in the base of tongue, suggestive of ectopic thyroid tissue (Figures 1 and 2). Laryngoscopy was performed and showed the presence of a mass at the base of the tongue with displacement of the uvula.

Thyroid hormone tests showed elevated thyroid-stimulating hormone (TSH = 10.79 (0.4–4.2)) concentrations and normal T4 concentrations (T4 = 0.81 (0.61–1.48)). From the clinical and imaging findings the diagnostic of ectopic thyroid and subclinical hypothyroidism were reached. The initial therapy was performed with Levothyroxine Sodium 25 mcg/day. Four months later a new evaluation was performed and there were no changes in the TSH levels. So, other exams were requested (anti-TPO antibodies 4 < 3.0 (normal value < 9.0); anti-thyroglobulin antibodies = 0.3 (normal value < 4.1)) to rule out the association between Hashimoto's thyroiditis and ectopic thyroid; however these tests showed normal values.

The dose of Levothyroxine Sodium was increased for 38 mcg/day. We observed no changes in the TSH after four months, showed in Table 1. Then the dose was increased again for 75 mcg/day. After four months a new TSH evaluation was performed and showed normal range.

The patient remains with thyroid hormone tests in normal range, and there are clinical improvement of the symptoms of dysphagia and foreign body sensation in the throat. Upon examination, there were no finds of a solid mass located at the base of the tongue. Therefore a surgical excision of the mass was not required; thus a follow-up protocol was established.

## 3. Discussion

Clinically, lingual thyroid is presented as a mass at the base of the tongue, pink and firm. The most important diagnostic tool is thyroid scan with technetium Tc-99m Sodium; computerized tomography and magnetic resonance imaging may help in defining the extension and location of the ectopic thyroid gland [6]. In this case report the diagnostic was obtained with aid of thyroid scintigraphy, ultrasound, and laryngoscopy.

Literature search showed only a few cases of lingual thyroid with hyperthyroidism and all of them were treated with a combination of antithyroid drugs, steroids, and surgery [7]. The case presents a subclinical hypothyroidism that receives treatment of regularization of TSH levels with Levothyroxine Sodium successfully.

The clinical presentation of lingual thyroid could be classified into two groups according to the appearance of the symptoms. In the first group of infants and young children, whose lingual thyroid is detected via routine screening may suffer from failure to thrive and mental retardation; or even severe respiratory distress and requiring emergency care [8, 9]. Other cases may present with onset of slowly progressing dysphagia and symptoms of oropharyngeal obstruction before or during puberty. This occurs as a response to the increased demand for thyroid hormone in these hypermetabolic states [8].

Treatment of a lingual thyroid depends on the sex and age of the patient as well as on the severity of the symptoms and the associated ulceration and hemorrhage. Patients with mild symptoms can be treated successfully by medical suppression, as presented in this paper [10]. However, most of cases received surgical treatment. The treatment with LT4 could result in partial involution of lingual thyroid volume.

Benign or malignant neoplastic changes were sometimes described in ectopic thyroid tissue [11]. Concerning these considerations, patients who are symptomatic need different therapeutic approaches depending on the localization of the gland, type of symptoms, malignancy of symptoms, and functional status [12].

The suppressive drug therapy based on suppressive dose of Levothyroxine depends on the ectopic gland size. This kind of approach is a good option for patients who are not suitable for surgery and have only obstructive symptoms. The patients who present a slow and progressive enlargement of the mass can receive this treatment and postpone the surgical procedure. Drug therapy based on Levothyroxine has been suggested to prevent malignant transformation of the ectopic gland and to prevent developing hypothyroidism [13]. In our case report the time required to regularize the levels of TSH was longer than usual; it could be related to time required to schedule new appointments and performed blood tests, once the patient was assisted in a public institution with a great workload in most hospitals and places.

## 4. Conclusion

Patients complaining of dysphagia should be evaluated to check whether there is a presence of ectopic thyroid. Examination of head and neck should have an especial attention at base of tongue. The presence of mass or abnormality in this area could be a sign of lingual thyroid ectopic, once this is the most affected region, and this place is the embryological origin of the thyroid. This case report highlights the management of this condition with drug therapy based on suppressive dose of Levothyroxine in a child.

## Figures and Tables

**Table 1:** Follow-up of laboratorial testing results related to treatment applied.

Period	Follow-up	Dosage of medication	TSH^*^
Initial		Without treatment	10.79 uUI/mL
First treatment	4 months	Levothyroxine Sodium 25 mcg/day^*^	10.1 uUI/mL
Second treatment	4 months	Levothyroxine Sodium 38 mcg/day	9.94 uUI/mL
Third treatment	4 months	Levothyroxine Sodium 50 mcg/day	10.9 uUI/mL
Final treatment		Levothyroxine Sodium 75 mcg/day	0.91 uUI/mL

^*^Normal TSH range 0.40–4.20** **uUI/mL.
